# Mechanism and Function of Antiviral RNA Interference in Mice

**DOI:** 10.1128/mBio.03278-19

**Published:** 2020-08-04

**Authors:** Qingxia Han, Gang Chen, Jinyan Wang, David Jee, Wan-Xiang Li, Eric C. Lai, Shou-Wei Ding

**Affiliations:** a Department of Microbiology and Plant Pathology, University of California, Riverside, Riverside, California, USA; b Department of Developmental Biology, Sloan Kettering Institute, New York, New York, USA; Icahn School of Medicine at Mount Sinai

**Keywords:** RNA interference, antiviral RNAi, interferons, plus-strand RNA virus, viral suppressor of RNAi

## Abstract

Distinct mammalian RNA viruses trigger Dicer-mediated production of virus-derived small-interfering RNAs (vsiRNA) and encode unrelated proteins to suppress vsiRNA biogenesis. However, the mechanism and function of the mammalian RNA interference (RNAi) response are poorly understood. Here, we characterized antiviral RNAi in a mouse model of infection with Nodamura virus (NoV), a mosquito-transmissible positive-strand RNA virus encoding a known double-stranded RNA (dsRNA)-binding viral suppressor of RNAi (VSR), the B2 protein. We show that inhibition of NoV RNA replication by antiviral RNAi in mouse embryonic fibroblasts (MEFs) requires Dicer-dependent vsiRNA biogenesis and Argonaute-2 slicer activity. We found that VSR-B2 of NoV enhances viral RNA replication in wild-type but not RNAi-defective MEFs such as Argonaute-2 catalytic-dead MEFs and Dicer or Argonaute-2 knockout MEFs, indicating that VSR-B2 acts mainly by suppressing antiviral RNAi in the differentiated murine cells. Consistently, VSR-B2 expression in MEFs has no detectable effect on the induction of interferon-stimulated genes or the activation of global RNA cleavages by RNase L. Moreover, we demonstrate that NoV infection of adult mice induces production of abundant vsiRNA active to guide RNA slicing by Argonaute-2. Notably, VSR-B2 suppresses the biogenesis of both vsiRNA and the slicing-competent vsiRNA-Argonaute-2 complex without detectable inhibition of Argonaute-2 slicing guided by endogenous microRNA, which dramatically enhances viral load and promotes lethal NoV infection in adult mice either intact or defective in the signaling by type I, II, and III interferons. Together, our findings suggest that the mouse RNAi response confers essential protective antiviral immunity in both the presence and absence of the interferon response.

## Abstract (precis)

Innate immune sensing of viral nucleic acids in mammals triggers potent antiviral responses regulated by interferons known to antagonize the induction of RNA interference (RNAi) by synthetic long double-stranded RNA (dsRNA). Here, we show that Nodamura virus (NoV) infection in adult mice activates processing of the viral dsRNA replicative intermediates into small interfering RNAs (siRNAs) active to guide RNA slicing by Argonaute-2. Genetic studies demonstrate that NoV RNA replication in mouse embryonic fibroblasts is inhibited by the RNAi pathway and enhanced by the B2 viral RNAi suppressor only in RNAi-competent cells. When B2 is rendered nonexpressing or nonfunctional, the resulting mutant viruses become nonpathogenic and are cleared in adult mice either intact or defective in the signaling by type I, II, and III interferons. Our findings suggest that mouse antiviral RNAi is active and necessary for the *in vivo* defense against viral infection in both the presence and absence of the interferon response.

## INTRODUCTION

A common antiviral response in mammals is the production of interferons (IFNs) triggered by the innate immune receptor sensing of the nonself viral nucleic acids (1–3). Engagement of IFN by IFN receptors induces STAT1- and STAT2-dependent transcription of numerous IFN-stimulated genes (ISGs) to establish an antiviral state. ISGs with known antiviral activities include those coding for 2′-5′-oligoadenylate synthetases (OAS) and double-stranded RNA (dsRNA)‐dependent protein kinase R (PKR) that are activated by cytosolic dsRNA. Subsequent RNase L activation and eukaryotic translation initiation factor 2 α (eIF2α) phosphorylation result in the degradation of viral and cellular RNAs and the inhibition of global cap-dependent protein translation, respectively (1–3).

Mammals harbor one Dicer for the biogenesis of both microRNAs (miRNAs) and small interfering RNAs (siRNAs) and four Argonautes, among which only Argonaute-2 (Ago2) retains the slicing activity essential for RNA interference (RNAi) (4–6). Recent studies have shown that infection of mammalian cells with six positive- and negative-strand RNA viruses from four families triggers Dicer recognition and processing of the viral dsRNA replicative intermediates, leading to production of abundant virus-derived siRNAs (7–9). Mammalian viral siRNAs (vsiRNAs) targeting these viruses are all highly enriched for 22-nucleotide (nt) canonical siRNA duplexes with 2-nt 3′ overhangs (10–15) and require Dicer for their biogenesis in mouse embryonic stem cells (mESCs) and human neural progenitor cells (hNPCs) as well as differentiated murine and human cells (11–14, 16). In counter defense, Nodamura virus (NoV; *Nodaviridae*), influenza A virus (IAV; *Orthomyxoviridae*), human enterovirus 71 (HEV71; *Picornaviridae*), and dengue virus-2 (DENV2; *Flaviviridae*) each encode a viral suppressor of RNAi (VSR), designated protein B2, NS1, 3A, and 2A, respectively. These VSRs share no primary sequence similarity, but all act to suppress Dicer processing of the vsiRNA precursors as dsRNA-binding proteins (10, 12–15). Thus, when VSR is rendered nonexpressing or nonfunctional, the resulting mutant viruses induce abundant vsiRNAs, replicate less efficiently than parental viruses in mESCs, mature murine, monkey, and human cells, and/or newborn mice and are efficiently rescued by knocking out all four Ago genes in mESCs or Dicer gene in human 293T cells (10, 12–15). Moreover, ebolavirus VP35 and the nucleocapsid protein of yellow fever virus (*Flaviviridae*), Semliki Forest virus (*Togaviridae*), and severe acute respiratory syndrome coronavirus (SARS CoV) and SARS CoV-2 also display activities of dsRNA-binding VSRs (15, 17–19, 64, 65). Together, these findings reveal a new mammalian antiviral response mediated by the RNAi pathway with striking similarities to the siRNA-directed antiviral response characterized extensively in plants and invertebrates (7–9, 20).

Several key questions remain unresolved on the mechanism and function of mammalian antiviral RNAi. For example, it is unknown whether viral infection induces *in vivo* production of vsiRNAs in adult mammals, which have an intact IFN response known to antagonize Dicer processing of artificial long dsRNA (21–25). It is also unknown whether vsiRNAs made in mammalian antiviral RNAi are *in vivo* loaded in the RNA-induced silencing complex (RISC) to guide specific RNA slicing by Ago2. In plants and insects, vsiRNA-RISC acts in the final step of antiviral RNAi as the effector complex so that Argonautes are dispensable for vsiRNA biogenesis (26–29). However, activation of the type I IFN (IFN-I) response by viral infection is inhibitory to miRNA-guided RNA slicing by Ago2 in cell culture (30), and there are contradictory reports on the antiviral activity of Ago2 in cultured cells (10–12, 16, 23, 31). Moreover, the validated mammalian VSRs are all dsRNA-binding proteins and include IAV NS1 and HEV71 3A, known to antagonize the IFN-I response (13, 32–34). Thus, it remains unclear whether suppression of RNAi by these dsRNA-binding VSRs plays an independent role in enhancing viral replication *in vitro* and *in vivo* (1–3, 35).

The understanding of new human antiviral immune responses has often depended on the mechanistic analysis in animal models of infection with well-characterized viruses. In this work, we examined the antiviral RNAi response of mice to the infection with NoV, which is mosquito transmissible and causes flaccid paralysis of the limbs and death in infant mice similarly to the infection with coxsackie viruses (36, 37). NoV contains two positive-strand genomic RNAs encoding three functional proteins in total and is a member of the *Nodaviridae* characterized extensively in viral RNA replication and antiviral RNAi (38–40). Nodaviral capsid protein is encoded by genomic RNA2. Nodaviral RNA1 codes for both the viral RNA replicase protein A and the VSR protein B2 and can self-replicate in the absence of RNA2 and produce the subgenomic RNA (RNA3) as the mRNA of VSR-B2 protein (38–40). We demonstrate that NoV RNA replication in adult mice induced production of abundant vsiRNAs active to guide specific RNA slicing by Ago2. We show that VSR-B2 inhibited production of both vsiRNAs and vsiRNA-RISC and became inactive to enhance NoV RNA replication in the absence of a functional RNAi pathway. Notably, B2 function is essential for robust NoV infection of adult mice either intact or defective in the interferon system. We propose that antiviral RNAi confers protective immunity against viral infection in adult mice.

## RESULTS

### Inhibition of viral RNA replication by antiviral RNAi requires Dicer-mediated vsiRNA biogenesis and Argonaute-2 slicer activity.

We first investigated the biogenesis and function of vsiRNAs in IFN-competent mouse embryonic fibroblasts (MEFs) commonly used to characterize innate immune antiviral responses (1–3, 35). Wild-type and RNAi-defective MEFs were transfected with transcripts of R1ΔB2, a mutant genomic RNA1 of NoV rendered defective in the translation of the B2 protein by three single-nucleotide substitutions (15, 41). At 3, 8, or 24 h posttransfection (hpt), the accumulation of the viral RNA1 and its subgenomic RNA (RNA3) synthesized after RNA1 self-replication was detected by Northern blotting or quantitative reverse transcription-PCR (RT-qPCR). The wild-type and RNAi-defective MEF lines were previously described (42), including Dicer-knockout (Dicer-KO) and Ago2-knockout (Ago2-KO) MEFs as well as Ago2 catalytic-dead MEFs (Ago2-CD) where Ago2 is expressed but is defective in RNA slicing due to substitution of the first aspartic acid in the DDH triad with an alanine (Ago2^D597A^).

**FIG 1:**
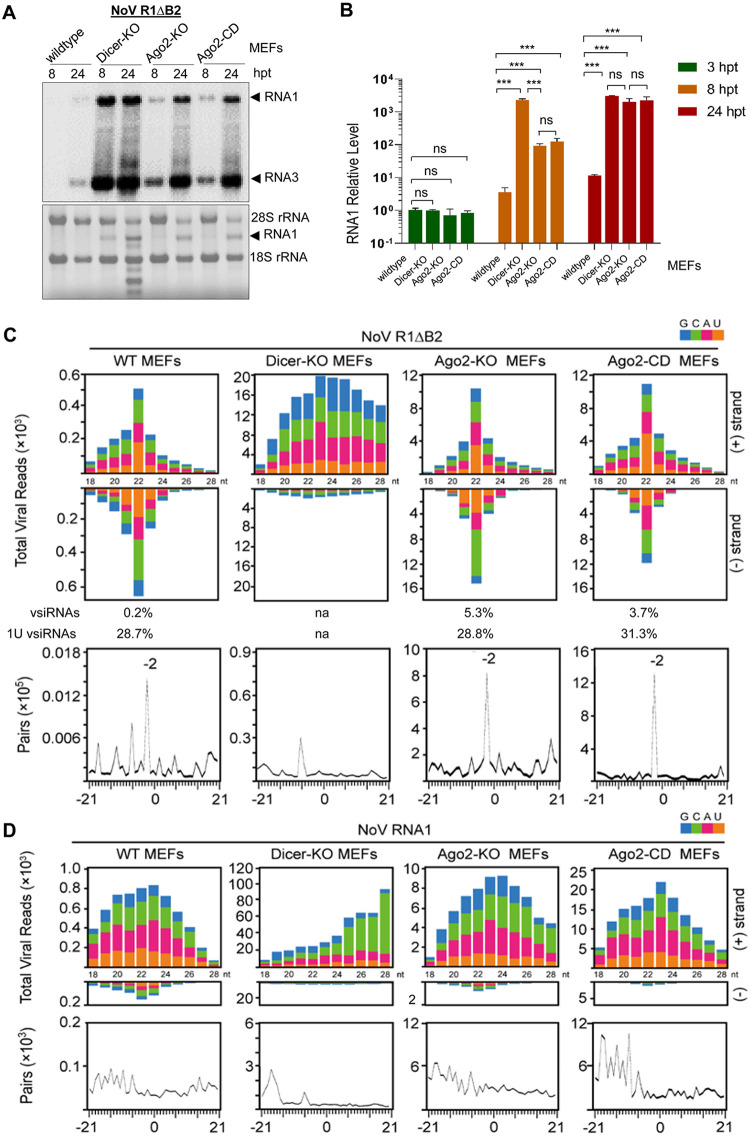
Dicer-mediated vsiRNA biogenesis and Ago2-dependent antiviral RNAi in differentiated murine cells. (A) Accumulation of NoV RNAs 1 and 3 detected by Northern blotting at 8 and 24 h posttransfection (hpt) of wild-type and homozygous Dicer-KO, Ago2-KO and Ago2-CD MEFs by electroporation with the same amounts of *in vitro* transcripts of NoV RNA1ΔB2 (R1ΔB2). Detection of the rRNAs served as loading controls. (B) Accumulation of the mutant viral RNA1 measured by RT-qPCR in the transfected MEFs at 3, 8, and 24 hpt and corrected by using β-actin mRNA as the internal reference. The results were from three independent experiments and are presented as means ± standard errors of the means (SEMs). A *t* test was used for statistical analysis. ***, *P* < 0.001; ns, not significant. Size distributions and abundances (shown per million of the total reads mapped to mouse and NoV genomes) of total virus reads from the four lines of MEFs at 24 hpt with R1ΔB2 (C) or wild-type NoV RNA1 (D). (C and D, bottom) The presence of pairs of 22-nt vsiRNA reads with 2-nt 3′ overhangs (−2 peak) by computing as described previously (15). The 5′-terminal nucleotide of virus reads is indicated by color. The abundance of vsiRNAs (21- to 23-nt) and 1U vsiRNAs, shown as percentage of the total mapped reads and total vsiRNAs, respectively, are given for those with a dominant population of vsiRNAs.

NoV R1ΔB2 replicated to levels detectable by Northern blotting in wild-type MEFs by 24 hpt but not at 8 hpt (Fig. 1A). In contrast, both the viral RNAs 1 and 3 were readily detectable at 8 hpt and reached extremely high levels visible by direct RNA staining by 24 hpt in all three lines of RNAi-defective MEFs (Fig. 1A). RT-qPCR analysis revealed that at 24 hpt, the viral RNA1 accumulated in the three lines of RNAi-defective MEFs at levels more than 100-fold higher than in wild-type MEFs (Fig. 1B). These results indicate that NoV RNA1 replication is significantly repressed in the differentiated MEFs by the RNAi pathway requiring not only Dicer and Ago2 but also the slicer activity of Ago2.

**FIG 2:**
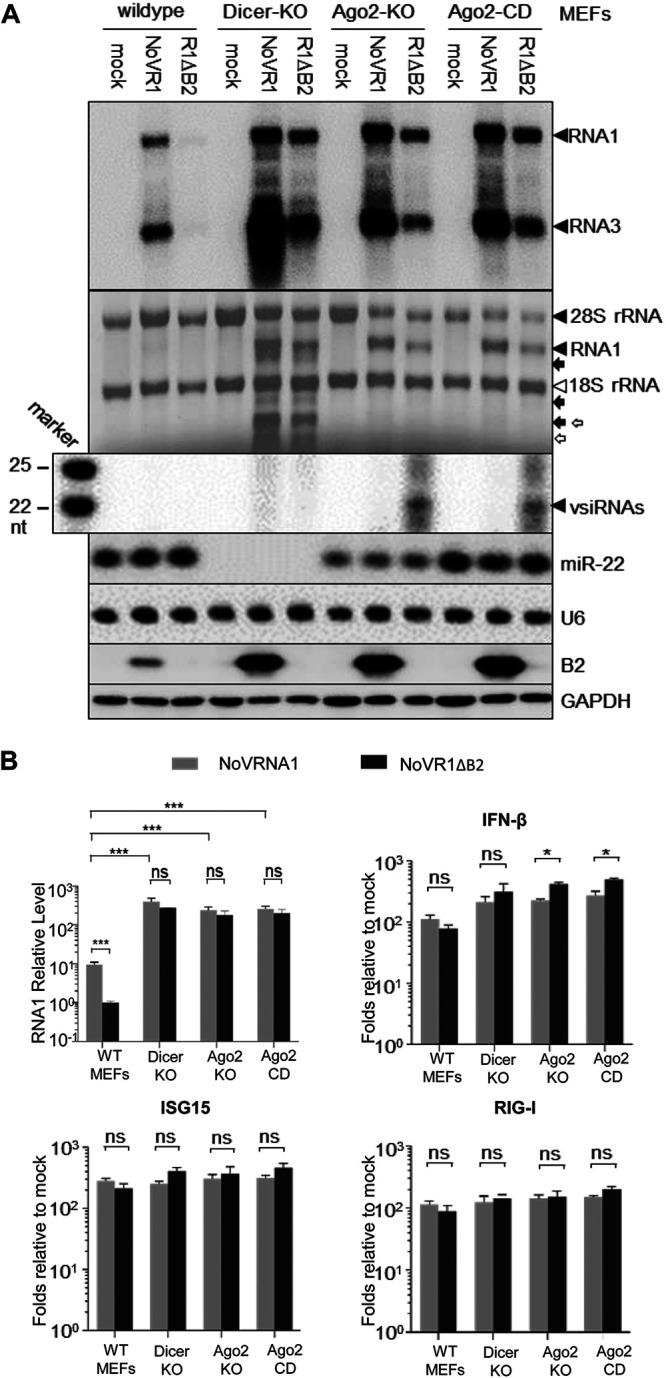
Function of dsRNA-binding VSR B2 protein in differentiated murine cells. (A) Northern or Western blot detection of viral RNAs 1 and 3, vsiRNAs, mouse miRNA 22-3p (miR-22), and the B2 VSR in the four lines of MEFs at 24 h posttransfection (hpt) with the same amounts of *in vitro* transcripts of wild-type NoV RNA1 or NoV RNA1ΔB2 (R1ΔB2). Detection of the rRNAs, U6 RNA, and glyceraldehyde 3-phosphate dehydrogenase (GAPDH) served as loading controls. Also shown is methylene blue staining of NoV RNA1 and the full-length and RNase L-cleaved fragments 28S and 18S rRNAs (indicated by solid and open arrows, respectively, according to the analysis by Northern blotting presented in Fig. S2 in the supplemental material). (B) Accumulation of the viral RNA1 (the abundance corrected using β-actin mRNA as the internal reference) and the mRNA of the IFN-β gene, *ISG15*, or *RIG-I* (fold change compared to mock transfection) detected by RT-qPCR in the MEFs 24 hpt with wild-type NoV RNA1 or R1ΔB2. The results were from three independent experiments and are presented as mean ± SEM.s *, *P* < 0.05, ***, *P* < 0.001, ns, not significant.

Deep sequencing of small RNAs from wild-type and RNAi-defective MEFs demonstrated that NoV RNA1 replication triggered production of a typical population of vsiRNAs not only in wild-type MEFs but also in Ago2-KO and Ago2-CD MEFs (Fig. 1C; see also Fig. S1A in the supplemental material). The 21- to 23-nt virus-derived small RNAs from wild-type, Ago2-KO, and Ago2-CD MEFs displayed approximately equal strand ratios with the 22-nt small RNAs as the most abundant and exhibiting strong enrichment for canonical siRNA duplexes with 2-nt 3′ overhangs (Fig. 1C; Fig. S1A). In contrast, the virus reads from Dicer-KO MEFs were predominantly positive strands and displayed no preference either in the size range of Dicer products or for canonical siRNA duplexes (Fig. 1C), suggesting loss of vsiRNA biogenesis in Dicer-KO MEFs. Consistently, we detected a marked reduction of mouse endogenous miRNAs in Dicer-KO MEFs compared to wild-type MEFs, but both Ago2-KO and Ago2-CD MEFs produced abundant endogenous miRNAs (see Fig. S2A and Table S1). These results indicate that the vsiRNAs detected in wild-type, Ago2-KO, and Ago2-CD MEFs were processed by Dicer from viral dsRNA precursors. Robust viral RNA replication in Ago2-KO and Ago2-CD MEFs induced production of more abundant vsiRNAs (Fig. 1C), readily detectable by Northern hybridization (Fig. 2A), than in wild-type MEFs. These findings indicate that in the differentiated murine cells, both the Dicer-mediated production of vsiRNAs and the slicer activity of Ago2 are essential for antiviral RNAi and that Ago2 is dispensable for the biogenesis of vsiRNAs.

10.1128/mBio.03278-19.1 TABLE S1 Contents and properties of the small RNA libraries. Download Table S1, DOCX file, 0.1 MB. Copyright © 2020 Han et al. 2020 Han et al. This content is distributed under the terms of the Creative Commons Attribution 4.0 International license.

10.1128/mBio.03278-19.5 FIG S1 Profiles of virus-derived siRNAs and small RNAs sequenced from wild-type and RNAi-defective MEFs after NoV RNA1 replication in the absence or presence of the B2 VSR. The data are from an additional set of libraries independent of those shown in Fig. 1C and D. Size distributions and abundances (shown per million of the total reads mapped to mouse and NoV genomes) of total virus reads from wild-type and homozygous Dicer-KO, Ago2-KO and Ago2-CD MEFs at 24 hpt with R1ΔB2 (A) or NoV RNA1 (B). (A and B, bottom) The presence of pairs of 22-nt vsiRNA reads with 2-nt 3′ overhangs (−2 peak) by computing as described previously (15). The 5′-terminal nucleotide of virus reads is indicated by color. The abundance of vsiRNAs (21- to 23-nt) and 1U vsiRNAs, shown as percentage of the total mapped reads and total vsiRNAs, respectively, are given for those with a dominant population of vsiRNAs. Download FIG S1, PDF file, 1.0 MB. Copyright © 2020 Han et al. 2020 Han et al. This content is distributed under the terms of the Creative Commons Attribution 4.0 International license.

10.1128/mBio.03278-19.6 FIG S2 Abundances and size distributions of mouse endogenous pre-miRNA hairpin reads in MEFs. The data are from the same sets of libraries shown in Fig. 1C and D. Size distributions and abundances (shown per million of the total reads mapped to mouse and NoV genomes) of host small RNA reads mapped to mouse pre-miRNA hairpin database from wild-type and homozygous Dicer-KO, Ago2-KO, and Ago2-CD MEFs at 24 hpt with R1ΔB2 (A) or NoV RNA1 (B). The 5′-terminal nucleotide of virus reads is indicated by color. Download FIG S2, PDF file, 0.5 MB. Copyright © 2020 Han et al. 2020 Han et al. This content is distributed under the terms of the Creative Commons Attribution 4.0 International license.

### The viral dsRNA-binding protein B2 enhances viral RNA replication in wild-type but not RNAi-defective mouse embryonic fibroblasts.

To analyze the function of dsRNA-binding VSR in differentiated cells, we compared NoV RNA1 replication in the presence or absence of B2 in wild-type and RNAi-defective MEFs. We measured the accumulation of the viral RNAs in MEFs by Northern blotting and RT-qPCR 24 h after transfection with the same amount of wild-type or B2-deficient NoV RNA1 (R1ΔB2) (Fig. 2). Known as mutant 1 (41), NoV R1ΔB2 contains the three single-nucleotide substitutions introduced into NoV RNA1 to eliminate the translational initiation from the first and second AUG codons of the B2 gene but alter neither the sequences of the viral replicase and the B1 protein encoded in the −1 reading frame of B2 nor the transcription of RNA3 (41, 43). Wild-type NoV RNA1 replicated to levels approximately 9-fold higher than R1ΔB2 in wild-type MEFs at 24 hpt (Fig. 2A and B). In contrast, we found no statistically significant differences between the accumulation levels of wild-type and VSR-deficient RNA1 in the three lines of RNAi-defective MEFs (Fig. 2A and B). Western blotting verified expression of VSR-B2 in all lines of MEFs after replication of wild-type, but not the mutant, NoV RNA1 (Fig. 2A). These genetic studies show that VSR-B2 enhanced viral RNA replication only in wild-type MEFs active in antiviral RNAi, indicating that the sole activity of VSR-B2 detectable in the differentiated murine cells is to suppress antiviral RNAi. Our findings are consistent with previous studies showing that B2 enhances the accumulation of viral RNA or protein in RNAi-competent cells (10, 15, 44–46) but not Saccharomyces cerevisiae (43), which lacks the RNAi pathway (47, 48).

Interestingly, wild-type NoV RNA1 replicated to significantly higher levels in all three lines of RNAi-defective MEFs than in wild-type MEFs, although the fold changes were smaller than that for NoV R1ΔB2 (Fig. 2A and B). These findings revealed the presence of active antiviral RNAi in wild-type MEFs that inhibited NoV RNA1 replication despite expression of VSR-B2, indicating that RNAi suppression by VSR-B2 is incomplete. Both Northern blotting (Fig. 2A) and deep sequencing (Fig. S1 and S2 and Table S1) found no obvious effect of B2 expression on the accumulation of mouse miRNAs in MEFs. In contrast to the vsiRNAs induced after NoV R1ΔB2 replication (Fig. 1C), the virus reads sequenced from wild-type, Ago2-KO, or Ago2-CD MEFs after replication of B2-expressing NoV RNA1 were predominantly positive strands and displayed no enrichment for canonical siRNA duplexes (Fig. 1D). Consistently, accumulation of vsiRNAs was not detectable by Northern blotting after replication of NoV RNA1 in the presence of VSR-B2, even though both wild-type and B2-deficient NoV RNAs replicated to similar levels in Ago2-KO and Ago2-CD MEFs (Fig. 2A and B). These results indicate that expression of VSR-B2 suppressed vsiRNA biogenesis in the differentiated murine cells. Nevertheless, we noted that unlike Dicer-KO MEFs, the low-abundant negative-strand virus reads from wild-type, Ago2-KO, or Ago2-CD MEFs after replication of NoV RNA1 in the presence of VSR-B2 exhibited the size distribution of vsiRNAs (Fig. 1D), suggesting that suppression of vsiRNA biogenesis by VSR-B2 is incomplete.

### Potent activation of the OAS/RNase L system by NoV RNA replication in the presence and absence of VSR-B2.

We further determined whether NoV RNA replication can trigger the IFN response in the immortalized MEFs. RT-qPCR analysis found that B2 expressed in *cis* from the replicating viral RNA1 in wild-type, Dicer-KO, Ago2-KO, or Ago2-CD MEFs had no significant effect on the induction of *ISG15* and *RIG-I* (Fig. 2B), two ISGs used frequently as the marker for the induction of the IFN response by RNA virus infection (1–3). B2 expression was associated with a modest decrease in the induction of the IFN-β gene in the three RNAi-defective MEFs but not wild-type MEFs (Fig. 2B). As described above (Fig. 2A and B), however, wild-type NoV RNA1 replicated to significantly higher levels than NoV R1ΔB2 in wild-type MEFs but not in Dicer-KO, Ago2-KO, or Ago2-CD MEFs, suggesting that the small increase in IFN-β gene expression was not inhibitory to viral RNA replication in RNAi-defective MEFs.

Strikingly, replication of both wild-type and B2-deficient NoV RNA1 induced strong RNase L-mediated cleavages of cellular rRNAs in Dicer-KO MEFs at 24 hpt but not 8 hpt (Fig. 1A and 2A; Fig. S3). These results show that NoV RNA1 replication potently activated the OAS/RNase L system in both the presence and absence of B2, indicating that abundant expression of the dsRNA-binding VSR-B2 in Dicer-KO MEFs is unable to prevent activation of the OAS/RNase L system. Dicer-KO MEFs accumulated highly abundant, positive-strand viral small RNAs with a wide size distribution during replication of wild-type and mutant NoV RNA1 (Fig. 1C and D), which may correspond to the derivatives of RNase L products. Intriguingly, similar RNase L activation was not observed not only in wild-type MEFs but also in the Ago2-KO and Ago2-CD MEFs that supported similarly robust replication of wild-type and mutant NoV RNA1 as in Dicer-KO MEFs (Fig. 1A and 2A; Fig. S3). Thus, the dramatically enhanced viral RNA replication alone in either the presence or absence of B2 was insufficient to ensure potent activation of the OAS/RNase L system. These findings suggest that activation of the OAS/RNase L system may be attenuated by Dicer processing or Dicer sequestration of viral dsRNA but not by VSR-B2 expression. Our findings together show that the dsRNA-binding VSR-B2 enhances viral RNA replication mainly by suppressing antiviral RNAi in the differentiated murine cells without major effects on the IFN response.

10.1128/mBio.03278-19.7 FIG S3 B2 VSR expression does not inhibit the activation of the IFN-inducible 2-5A/RNase L system triggered by NoV RNA replication. (A) Diagrams showing RNase L cleavage sites (black arrows) and the regions of 28S and 18S rRNAs targeted by the 5′ and 3′ terminal probes in Northern blot analysis. (B) Northern blot detection of the full-length and RNase L-cleaved fragments of 28S (top) and 18S (bottom) rRNAs by the 5′- and 3′-terminal probes from wild-type and homozygous Dicer-KO, Ago2-KO, and Ago2-CD MEFs 24 h posttransfection (hpt) with buffer (mock) or the same amounts of *in vitro* transcripts of NoV wild-type RNA1 or RNA1ΔB2 (R1ΔB2). Ethidium bromide staining (left, see also Fig. 1A and 2A) detected the viral RNAs 1 and 3 (black arrowheads), 28S (light gray arrowheads)/18S (dark gray arrowheads) rRNAs and several additional RNA bands from Dicer-KO MEFs after NoV RNA1 replication in both the absence or presence of B2, but not after mock transfection. Northern blot analysis showed that these additional RNA bands corresponded to 28S rRNA fragments of approximately 2.5, 1.5, and 1.0 kb (dark gray arrows) and 18S rRNA fragments of approximately 1.0 and 0.7 kb (light gray arrows), respectively. Accumulation of the 1.5- and 1.0-kb fragments of 28S rRNA and of the 1.0- and 0.7-kb fragments of 18S rRNAs was also detected at lower levels in Ago2-KO and Ago2-CD MEFs after NoV RNA1 replication, in either the absence or presence of B2, but not after mock transfection. However, these RNase L-mediated cleavages of 28S and 18S rRNAs were not detected in wild-type MEFs either mock transfected or transfected with NoV wild-type RNA1 or R1ΔB2. Our results indicate that enhanced NoV RNA replication in RNAi-defective MEFs triggers activation of the IFN-inducible 2-5A/RNase L, which is not inhibited by the B2 VSR expressed at high levels in these MEFs. Download FIG S3, PDF file, 1.0 MB. Copyright © 2020 Han et al. 2020 Han et al. This content is distributed under the terms of the Creative Commons Attribution 4.0 International license.

### Production and Argonaute loading of abundant vsiRNAs in adult mice with an intact IFN system.

**FIG 3:**
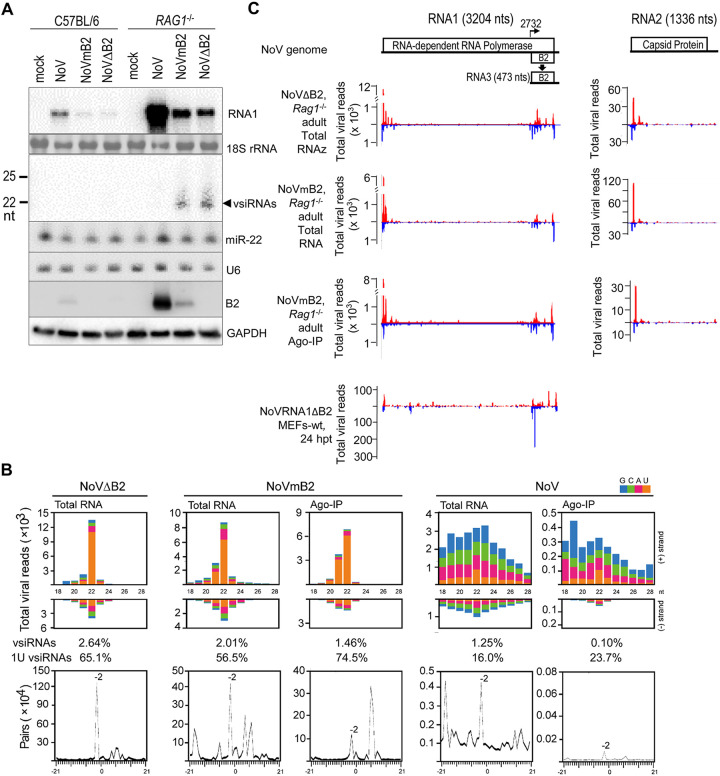
Potent induction of antiviral RNAi in adult mice with an intact IFN system. (A) Northern or Western blot detection of viral RNA1, vsiRNAs, mouse miRNA 22-3p (miR-22), and the B2 VSR in the hind limb skeletal muscle tissues of adult mice at 5 dpi with buffer (mock) or the same amount of NoV, NoVmB2, or NoVΔB2. Detection of 18S rRNA, U6 RNA, and GAPDH served as loading controls. (B) Size distributions and abundance (shown per million of the total reads mapped to mouse and NoV genomes) of the total and Argonaute-bound virus reads sequenced from *Rag1*^−/−^ adult mice 5 days postinfection (dpi) by intraperitoneal injection with the same amounts of NoVΔB2, NoVmB2, or NoV. (Bottom) The presence of pairs of 22-nt vsiRNA reads with 2-nt 3′ overhangs (−2 peak) by computing as described previously (15). The 5′-terminal nucleotide of virus reads is indicated by color. The abundances of vsiRNAs (21- to 23-nt) and 1U vsiRNAs, shown as percentage of the total mapped reads and total vsiRNAs, respectively, are given. (C) Virus genome distribution of the total and Argonaute-bound 21- to 23-nt vsiRNAs (per million of total mapped reads) from *Rag1*^−/−^ adult mice infected with NoVΔB2 or NoVmB2 or from wild-type MEFs after NoV RNA1 replication. The functional proteins encoded by the viral bipartite RNA genome and transcription of B2 mRNA (RNA3) from RNA1 are shown.

We next explored whether antiviral RNAi is induced by viral infection in adult mice (6 to 8 weeks old), which are known to activate more-potent IFN responses than in cultured cells or infant mice (1–3, 35). We found that after intraperitoneal injection, wild-type NoV, NoVΔB2, and NoVmB2 all replicated to markedly lower levels in the limb muscular tissues of wild-type adult mice (C57BL/6) than in mutant mice knocked out of recombination activating gene 1 (*Rag1^−/−^*) (Fig. 3A), which lack mature B and T lymphocytes to direct adaptive immunity but have an intact IFN system (49). Whereas B2 is rendered nonexpressing in NoVΔB2, NoVmB2 differs from NoV by a single nucleotide in RNA1 and expresses a mutant B2 protein defective in dsRNA binding and RNAi suppression, but the introduced mutation does not alter the amino acid sequence of the viral replicase and the B1 protein encoded in the −1 reading frame of B2 (15, 26).

Deep sequencing of total small RNAs showed that in addition to the endogenous miRNAs (see Fig. S4 and Table S1), *Rag1^−/−^* mice produced a typical population of mammalian vsiRNAs in response to the infection with either NoVΔB2 or NoVmB2 (Fig. 3B). Most of the virus reads cloned from the limb tissues of NoVΔB2- or NoVmB2-infected *Rag1^−/−^* mice at 5 days postinfection (dpi) were in the 21- to 23-nt size range of Dicer products, among which the 22-nt size species was the most abundant for both the positive and negative strands and exhibited strong enrichment for canonical siRNA duplexes with 2-nt 3′ overhangs (Fig. 3B; Table S1). Notably, the vsiRNAs from *Rag1^−/−^* mice infected with either NoVΔB2 or NoVmB2 were readily detectable by Northern blotting (Fig. 3A). The total small RNA reads in NoVΔB2 and NoVmB2 libraries that mapped to the viral and mouse genomes contained 2.01% to 2.64% vsiRNAs in the 21- to 23-nt size range (Fig. 3B; Table S1), which were more abundant than those (0.04% to 0.59%) reported in cultured mammalian cells, newborn mice, or adult flies (20).

10.1128/mBio.03278-19.8 FIG S4 Abundances and size distributions of mouse endogenous pre-miRNA hairpin reads in *Rag1*^−/−^ adult mice. The data are from the same sets of libraries shown in Fig. 3B. Size distributions and abundances (shown per million of the total reads mapped to mouse and NoV genomes) of host small RNA reads mapped to mouse pre-miRNA hairpin database from *Rag1*^−/−^ adult mice 5 days postinfection (dpi) by intraperitoneal injections with the same amounts of NoVΔB2, NoVmB2, or NoV. The 5′-terminal nucleotide of virus reads is indicated by color. Download FIG S4, PDF file, 0.3 MB. Copyright © 2020 Han et al. 2020 Han et al. This content is distributed under the terms of the Creative Commons Attribution 4.0 International license.

To date, Ago-loaded mammalian vsiRNAs have been sequenced only in cell culture (20). We found abundant vsiRNAs in the immunoprecipitants obtained with a pan-Ago antibody from NoVmB2-infected *Rag1^−/−^* mice (Fig. 3B), indicating *in vivo* Argonaute loading of mouse vsiRNAs. Of note, adult mouse vsiRNAs (Fig. 3B) exhibited strong preference for uracil as the 5′-terminal nucleotide (1U), and these 1U-vsiRNAs were further enriched in Argonaute immunoprecipitants (Fig. 3B; Table S1), similarly to endogenous miRNAs (50) and influenza vsiRNAs sequenced from cell culture (12). In support of selective Argonaute loading of vsiRNAs, we found that Ago-bound vsiRNAs were de-enriched for canonical siRNA duplexes compared to the total vsiRNAs (Fig. 3B). By comparison, virus genome distribution patterns of the vsiRNA hot spots were more similar between the total and the Argonaute-bound populations sequenced from NoVmB2-infected *Rag1^−/−^* mice than between the vsiRNAs produced in MEFs and adult mice (Fig. 3C). Together, these results demonstrate efficient Dicer processing of the viral dsRNA and subsequent loading of the resulting vsiRNAs into RISC in the infected adult mice with an intact IFN system.

Both Northern blotting (Fig. 3A) and deep sequencing (Table S1) found no obvious differences in the accumulation of mouse miRNAs in *Rag1^−/−^* mice without or with the infection by NoVΔB2, NoVmB2, or NoV. NoV infection of *Rag1^−/−^* mice in the presence of B2 induced a detectable population of 22-nt vsiRNA duplexes with 2-nt 3′ overhangs (Fig. 3B). However, these vsiRNAs were low in abundance and undetectable by Northern hybridization in contrast to those in mice infected with NoVmB2 or NoVΔB2 (Fig. 3A and B; Table S1). Moreover, the virus reads found in NoV-infected *Rag1^−/−^* adult mice exhibited a strong positive-strand bias, and only the negative strands exhibited a weak size preference for 22 nt (Fig. 3B). Highly abundant endogenous miRNAs accumulated in Argonaute immunoprecipitants from both NoV- and NoVmB2-infected *Rag1^−/−^* mice (Fig. S4 and Table S1). Compared to that with NoVmB2 infection, however, Argonaute immunoprecipitants from NoV-infected mice contained much-less-abundant virus reads, and only the negative strands in the immunoprecipitants showed an obvious size preference for Dicer products (Fig. 3B). In addition, 1U enrichment was visible for neither the total nor Argonaute-bound virus reads from NoV-infected mice (Fig. 3B). These findings indicate that in *Rag1^−/−^* mice, expression of VSR-B2 interfered with the biogenesis of vsiRNAs, but not the endogenous miRNAs, similar to the findings in MEFs (Fig. 1 and 2).

### Viral infection of *Rag1^−/−^* mice induces production of vsiRNA-RISC active to direct specific RNA slicing by Ago2.

**FIG 4:**
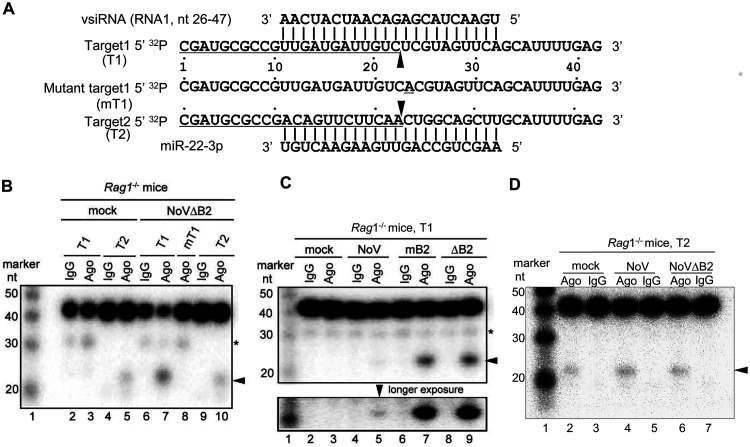
RNA slicing-competent Ago2-RISC from healthy and infected *Rag1*^−/−^ adult mice. (A) The nucleotide sequences of synthetic single-stranded RNAs T1 and T2 labeled at the 5′ terminus by ^32^P to serve as the *in vitro* slicing target guided by a cloned vsiRNA (corresponding to nucleotides 26 to 47 of NoV RNA1) and mouse miR-22-3p, respectively. The expected slicing sites between the nucleotides base paired with the 5′-terminal 10th and 11th nucleotides of the vsiRNA or miR-22 are marked by an arrowhead, and the resulting 5′-terminal ^32^P-labeled slicing products (22 nucleotides in length) are underlined. An introduced U→A single nucleotide substitution into the vsiRNA target (T1) disrupts the base pairing between the 10th nucleotide of the vsiRNA with its target (mT1, residue A underlined) known to be essential for slicing. (B to D) *In vitro* slicing assay using pan-Ago or mouse control IgG immunoprecipitants from total extracts of hind limb skeletal muscle tissues of *Rag1*^−/−^ adult mice 5 days postinoculation with buffer (mock) or the same amounts of NoV, NoVmB2, or NoVΔB2. The positions of the 22-nt 5′ cleavage products are indicated by arrowheads on the right. One fragment (∼31 nucleotides) of T1 and mT1 resulting from an unknown cleavage event is marked by *. A longer exposure is shown at the bottom of panel C.

Dicer processing of the viral dsRNA replicative intermediates produces multiple overlapping sets of vsiRNAs in the infected cells (26, 51) (Fig. 3C), making it difficult to map Ago2-RISC slicing of the viral RNA guided by individual vsiRNAs. Thus, we designed an *in vitro* slicing assay using three synthetic single-stranded RNAs as the slicing target (Fig. 4A) to determine whether the vsiRNAs made by adult mice in response to viral infection are active to guide specific RNA slicing by Ago2 in RISC. Each target RNA contained a central region complementary to a single vsiRNA or mouse endogenous miRNA 22 (miR-22) known to accumulate in the pan-Ago immunoprecipitants (IP) after *in vivo* infection with NoVΔB2 (Fig. 3A) and thus avoided the targeting by multiple vsiRNAs produced after *in vivo* infection. We detected the expected 5′ cleavage product of 22 nucleotides long after incubation of T2, the target RNA of miR-22, with pan-Ago IP from both the mock- and NoVΔB2-infected *Rag1^−/−^* adult mice (Fig. 4B, lanes 5 and 10) but not with IP using the control IgG from the same mice (Fig. 4B, lanes 4 and 9). These findings indicate that miR-22-RISC isolated from both the mock- and NoVΔB2-infected adult mice was active in RNA slicing by Ago2.

We found that the vsiRNA target (T1) was efficiently cleaved by the pan-Ago IP from NoVΔB2-infected *Rag1^−/−^* mice, yielding the predicted 22-nt 5′ cleavage product (Fig. 4B, lane 7; Fig. 4C, lane 9). However, the control IgG IP from the same mice was inactive in the specific slicing of T1 (Fig. 4B, lane 6; Fig. 4C, lane 8). Unlike the slicing of the miR-22 target, neither the control IgG IP nor the pan-Ago IP from the mock-infected *Rag1^−/−^* mice was active in the slicing of the vsiRNA target (Fig. 4B and C, lanes 2 and 3). Moreover, the pan-Ago IP from NoVΔB2-infected *Rag1^−/−^* mice became inactive in slicing mT1 RNA, which contained a single nucleotide mutation to disrupt the base pairing of the vsiRNA target with the 10th nucleotide of the vsiRNA (Fig. 4A and B, lane 8), known to be required for Ago2 slicing of RNAs targeted by an siRNA in mammalian RNAi (5, 6). These results show that NoVΔB2 infection triggered production of vsiRNA-RISC active to direct specific RNA slicing by Ago2 and was not inhibitory to Ago2 slicing programmed by endogenous miRNA in *Rag1^−/−^* adult mice.

### Expression of a functional VSR-B2 inhibits *in vivo* production of slicing-competent RISC programmed by vsiRNA but not endogenous miRNA.

We next determined whether B2 expression *in vivo* interferes with Ago2 slicing guided by vsiRNA or miR-22. To this end, we compared T1/T2 RNA slicing by the control and the pan-Ago IP isolated from *Rag1^−/−^* mice after infection with the three strains of NoV characterized above in the ability to induce production of vsiRNAs. When the vsiRNA target T1 was incubated with the pan-Ago IP from NoV-infected *Rag1^−/−^* mice, the 22-nt 5′ cleavage product was detectable only after longer exposure (Fig. 4C, lane 5, bottom), unlike those from NoVΔB2-infected *Rag1^−/−^* mice. In contrast, no obvious difference was observed in the slicing of the vsiRNA target by the pan-Ago IP isolated from *Rag1^−/−^* mice infected with either NoVΔB2 or NoVmB2 (Fig. 4C, compare lanes 7 and 9), indicating that unlike wild-type B2, the mutant B2 expressed by NoVmB2 was not inhibitory to the production of the slicing-competent vsiRNA-RISC. However, the slicing of the miR-22 target was similar after incubation with the pan-Ago IP isolated from *Rag1^−/−^* mice after mock infection and infection with either NoVΔB2 or NoV (Fig. 4D, lanes 2, 4, and 6). These findings indicate that expression of an RNAi suppression-competent B2 protein from NoV inhibits the production of slicing-competent Ago2-RISC programmed by vsiRNA but not by endogenous miRNA.

### Expression of a functional VSR-B2 is essential for high load and lethality of NoV in adult mice intact or defective in the IFN system.

**FIG 5:**
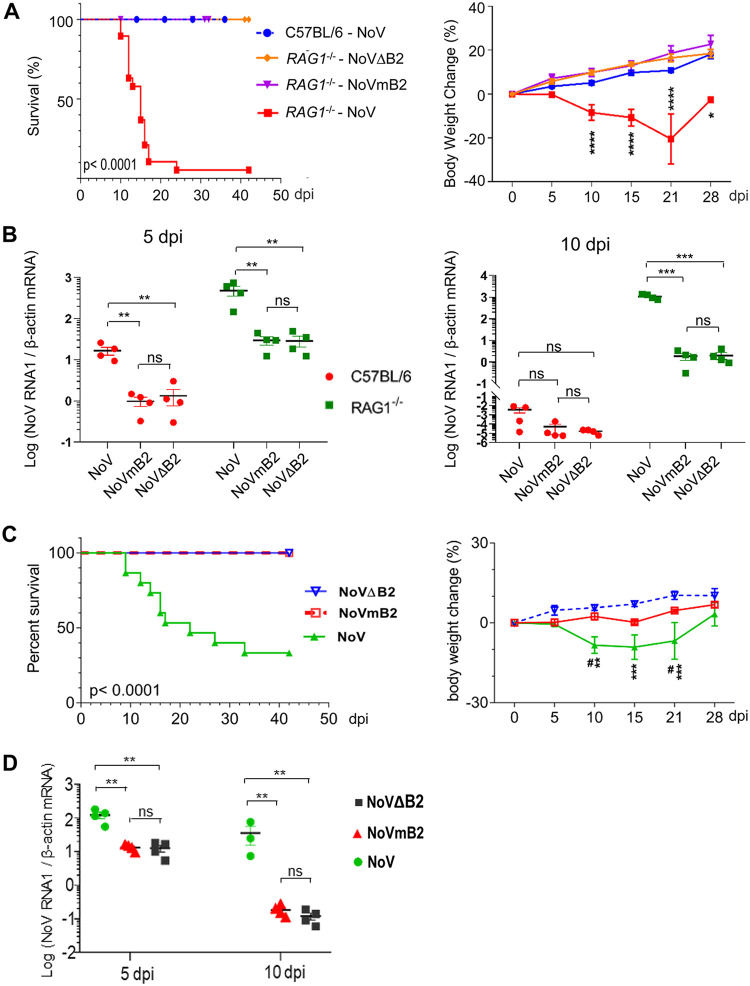
Expression of a functional VSR enhances virus load and promotes lethal NoV infection in adult mice intact or defective in the IFN system. (A) Survival (left) and body weight changes (right) of wild-type C57BL/6 or *Rag1*^−/−^ adult mice after infection by intraperitoneal injections with the same amounts of NoV, NoVΔB2, or NoVmB2. The infected mice used for survival analysis were C57BL/6 (NoV, *n* = 18) and *Rag1*^−/−^ (NoV, *n* = 19; NoVΔB2, *n* = 15; NoVmB2, *n* = 18) and for body weight analysis were C57BL/6 (NoV, *n* = 15) and *Rag1*^−/−^(NoV, *n* = 15; NoVΔB2, *n* = 15; NoVmB2, *n* = 10). (B) The viral titers of NoV, NoVΔB2, and NoVmB2 in mouse hind limb skeletal muscle tissues of C57BL/6 or *Rag1*^−/−^ adult mice detected at 5 and 10 days postinfection (dpi) by RT-qPCR of the viral RNA1 using β-actin mRNA as the internal reference. (C) Survival (left) and body weight changes (right) of *STAT1* and *STAT2* double-knockout adult mice (*Stat1/2*^−/−^) after infection with the same amount of NoV (*n* = 15), NoVΔB2 (*n* = 6), or NoVmB2 (*n* = 5). (D) The virus titers of NoV, NoVΔB2, and NoVmB2 in *Stat1/2*^−/−^ adult mouse hind limb skeletal muscle tissues detected at 5 and 10 days postinfection (dpi) by RT-qPCR of the viral RNA1 using β-actin mRNA as the internal reference. Values of individual mice and the means ± SEMs are presented. *, *P* < 0.05; **, *P* < 0.01; ***, *P* < 0.001; ****, *P* < 0.0001; ns, not significant.

Wild-type C57BL/6 adult mice displayed no signs of disease after NoV infection (Fig. 5A), as reported previously for the inoculation of BALB/c mice 21 days after birth or older (36, 37). In contrast, 95% of *Rag1^−/−^* adult mice from independent experiments succumbed within 25 days postinfection with NoV, and the infected mice exhibited significant weight loss (Fig. 5A), indicating a protective role of adaptive immunity in adult mice against NoV. Notably, NoVΔB2 induced no weight loss or any other signs of disease up to 42 dpi in the inoculated *Rag1^−/−^* adult mice (Fig. 5A). *Rag1^−/−^* mice also exhibited no signs of disease or weight loss after inoculation with NoVmB2 (Fig. 5A). All of the three viruses accumulated to lower levels in C57BL/6 mice than in *Rag1^−/−^* mice at 5 dpi and were largely cleared in C57BL/6 mice by 10 dpi (Fig. 3A and 5B). B2 was not essential for the production of infectious virions, as virion preparations from NoV-, NoVΔB2-, or NoVmB2-infected *Rag1^−/−^* adult mice were all able to induce systemic infection in newborn C57BL/6 mice, in contrast to Flock house virus replication in nonhost hamster cells (45). At 10 dpi in *Rag1^−/−^* mice, NoV titers were approximately 500 times higher than either NoVΔB2 or NoVmB2 (Fig. 5B). These findings show that expression of a functional VSR-B2 was required for the high load and lethality of NoV in *Rag1^−/−^* adult mice with an intact IFN system.

**FIG 6:**
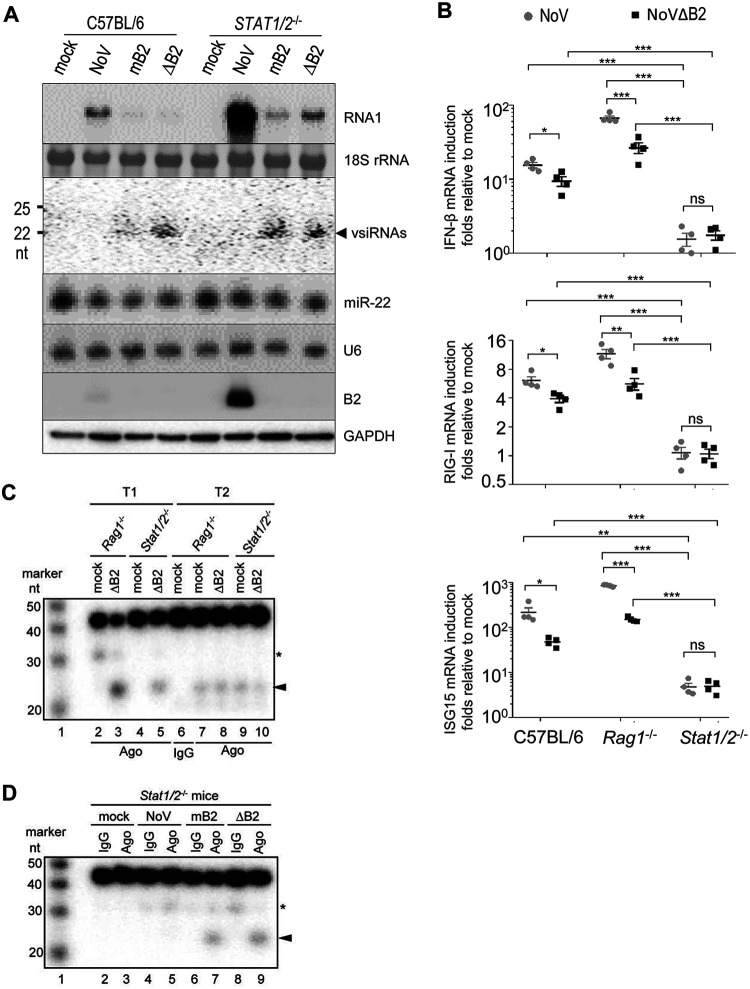
Induction and suppression of antiviral RNAi in *Stat1/2*^−/−^ adult mice. (A) Northern or Western blot detection of the viral RNA1, vsiRNAs, mouse miRNA 22-3p (miR-22), and the B2 VSR in the hind limb skeletal muscle tissues of C57BL/6 and *Stat1/2*^−/−^ adult mice at 5 dpi with buffer (mock) or the same amounts of NoV, NoVmB2, or NoVΔB2. Detection of 18S rRNA, U6 RNA, and GAPDH served as loading controls. (B) Fold changes of the IFN-β gene (top), *RIG-I* (middle), and *ISG15* (bottom) mRNAs detected by RT-qPCR in mouse hind limb skeletal muscle tissues of C57BL/6, *Rag1*^−/−^, or *Stat1/2*^−/−^ adult mice at 5 dpi with the same amounts of NoV, NoVmB2, or NoVΔB2 relative to that with mock infection. (C and D) *In vitro* slicing assay using pan-Ago or mouse control IgG immunoprecipitants from total extracts of hind limb skeletal muscle tissues of *Rag1*^−/−^ or *Stat1/2* ^−/−^ adult mice 5 days postinoculation with buffer (mock) or the same amounts of NoV, NoVmB2, or NoVΔB2. The positions of the 22-nt 5′ cleavage products are indicated by arrowheads on the right. One fragment (∼31 nucleotides) of T1 and mT1 resulting from an unknown cleavage event is marked by *. Values of individual mice and the means ± SEMs are presented. *, *P* < 0.05; **, *P* < 0.01; ***, *P* < 0.001; ns, not significant.

We further compared NoV, NoVΔB2, and NoVmB2 infection in *STAT1* and *STAT2* double-knockout mice (*Stat1/2^−/−^*), which are defective in the signaling by type I, II, and III interferons (2, 3). The results showed that *Stat1/2^−/−^* adult mice also were highly susceptible to NoV and 60% of the *Stat1/2^−/−^* mice succumbed within 30 days of infection with NoV, which was accompanied with significant weight loss (Fig. 5C). However, neither NoVΔB2 nor NoVmB2 induced weight loss or any other signs of disease up to 42 dpi in *Stat1/2^−/−^* adult mice (Fig. 5C). RT-qPCR (Fig. 5D) and Northern blotting (Fig. 6A) revealed systemic spread of all three viruses to the limb muscular tissues of *Stat1/2^−/−^* mice after intraperitoneal injection. However, whereas both NoVΔB2 and NoVmB2 were largely cleared by 10 dpi, NoV titers remained high in the infected *Stat1/2^−/−^* mice at 10 dpi (Fig. 5D). These results indicate that expression of a functional VSR-B2 was essential to inhibit the clearance of NoV and induce lethality in adult mice defective in IFN signaling.

### The IFN response is not inhibitory to *in vivo* production of vsiRNAs or slicing-competent vsiRNA-RISC.

RT-qPCR analysis revealed that the IFN-β gene, *RIG-I*, and *ISG15* were all induced by infection with both NoV and NoVΔB2 in C57BL/6 and *Rag1^−/−^* mice compared to that with the infection of *Stat1/2^−/−^* mice (Fig. 6B). By comparison, IFN-β, *RIG-I*, and *ISG15* mRNAs accumulated to higher levels in C57BL/6 and *Rag1^−/−^* adult mice after the infection with NoV than with NoVΔB2 (Fig. 6B), indicating that expression of VSR-B2 from NoV did not inhibit the induction of the IFN response in adult mice, consistent with the findings from MEFs (Fig. 2).

Northern blot analysis showed that infection with either NoVΔB2 or NoVmB2 induced production of vsiRNAs in *Stat1/2^−/−^* adult mice at levels comparable to that in C57BL/6 mice (Fig. 6A), which accumulated markedly reduced levels of vsiRNAs compared to that in *Rag1^−/−^* mice (Fig. 3A). Similarly to that with NoV infection of *Rag1^−/−^* mice (Fig. 3A), vsiRNAs were undetectable by Northern blotting in NoV-infected *Stat1/2^−/−^* mice (Fig. 6A). Consistent with the results of Northern blotting, deep sequencing of total small RNAs revealed production of a typical vsiRNA population by C57BL/6 and *Stat1/2^−/−^* mice in response to NoVΔB2 infection (see Fig. S5 and Table S1). Low-abundant negative-strand 22-nt vsiRNAs strongly enriched for canonical siRNA duplexes with 2-nt 3′ overhangs were also visible in NoV-infected C57BL/6 and *Stat1/2^−/−^* mice (Fig. S5), as was found in NoV-infected *Rag1^−/−^* mice (Fig. 3B). Moreover, we detected active slicing of the vsiRNA target (T1) by the pan-Ago IP from *Stat1/2^−/−^* mice infected with either NoVΔB2 or NoVmB2, but not those from mock-infected *Stat1/2^−/−^* mice (Fig. 6C, lane 5; Fig. 6D, lanes 7 and 9). These findings show that viral infection of IFN-defective *Stat1/2^−/−^* mice induced production of not only vsiRNAs at levels detectable by Northern blotting, but also vsiRNA-RISC active to direct specific RNA slicing by Ago2. However, neither the control nor the pan-Ago IP from NoV-infected *Stat1/2^−/−^* mice directed detectable cleavage of the vsiRNA target (Fig. 6D, lanes 4 and 5), indicating that expression of a functional VSR-B2 inhibits production of slicing-competent vsiRNA-RISC in *Stat1/2^−/−^* mice.

10.1128/mBio.03278-19.9 FIG S5 Size distributions and abundances (shown per million of the total reads mapped to mouse and NoV genomes) of the total virus reads sequenced from wild-type C57BL/6 (A) and *Stat1/2*^−/−^ (B) adult mice 5 days postinfection (dpi) by intraperitoneal injections with the same amounts of NoVΔB2 or NoV. (A and B, bottom) The presence of pairs of 22-nt vsiRNA reads with 2-nt 3′ overhangs (−2 peak) by computing as described previously (15). The 5′-terminal nucleotide of virus reads is indicated by color. We noted that the total small RNA reads in both of the NoVΔB2 libraries that mapped to the viral and mouse genomes contained approximately 0.04% vsiRNAs in the 21- to 23-nt size range (Table S1), even though NoVΔB2 replicated to higher levels in *Stat1/2*^−/−^ mice than in C57BL/6 mice (Fig. 6A). This suggests that the viral dsRNA may be processed by Dicer into vsiRNAs less efficiently in *Stat1/2*^−/−^ mice than in C57BL/6 mice. Download FIG S5, PDF file, 0.7 MB. Copyright © 2020 Han et al. 2020 Han et al. This content is distributed under the terms of the Creative Commons Attribution 4.0 International license.

We noted weaker slicing of the vsiRNA target by the pan-Ago IP from *Stat1/2^−/−^* mice than from *Rag1^−/−^* mice in response to NoVΔB2 infection (Fig. 6C, compare lanes 3 and 5), which appeared to correlate with the lower levels of vsiRNAs induced by NoVΔB2 in *Stat1/2^−/−^* mice than in *Rag1^−/−^* mice (Fig. 3A and 6A). However, no obvious differences were observed in the slicing of the miR-22 target by the pan-Ago IP from either *Rag1^−/−^* or *Stat1/2^−/−^* mice after mock or NoVΔB2 infection (Fig. 6C, compare lanes 7 to 9). These findings together indicate that active STAT1/STAT2-dependent IFN signaling in *Rag1^−/−^* adult mice was not inhibitory to the production of vsiRNAs or slicing-competent Ago2-RISC programmed by either vsiRNA or endogenous miRNA.

## DISCUSSION

Distinct positive- and negative-strand RNA viruses from the *Flaviviridae*, *Nodaviridae*, *Orthomyxoviridae*, and *Picornaviridae* induce Dicer-mediated production of vsiRNAs and encode unrelated dsRNA-binding VSRs to suppress the biogenesis of cognate vsiRNAs in mammalian cells (10, 12–15). Results from this work provide several important insights into the mechanism and function of the new mammalian antiviral response.

Early studies, including those characterizing induction of RNAi by artificial long dsRNA, suggested inhibition of Dicer-mediated biogenesis of vsiRNAs by the IFN response (8, 10). Here, we demonstrate that when VSR-B2 was rendered nonexpressing or nonfunctional, NoV RNA replication triggered the production of highly abundant vsiRNAs not only in IFN-competent MEFs but also in *Rag1^−/−^* adult mice with an intact IFN system. The mouse vsiRNAs made in both MEFs and adult mice were highly enriched for 22-nt siRNA duplexes with 2-nt 3′ overhangs, indicating that they are processed by Dicer from viral dsRNA precursors. Consistently, we show that the production of vsiRNAs in MEFs was undetectable in Dicer-KO MEFs. Moreover, *Ago2* was dispensable for vsiRNA biogenesis in the differentiated murine cells. Deep sequencing of total small RNAs in pan-Argonaute immunoprecipitants from NoVmB2-infected *Rag1^−/−^* mice illustrated that vsiRNAs were *in vivo* loaded in RISC. Similarly to endogenous miRNAs, 1U-vsiRNAs were enriched in NoVmB2-infected *Rag1^−/−^* mice, especially in Argonaute immunoprecipitants, and the selective vsiRNA loading may explain why Argonaute-bound vsiRNAs from adult mice were de-enriched for vsiRNA duplexes. Notably, Northern blot detection of vsiRNAs in NoVmB2- or NoVΔB2-infected adult mice revealed no enhanced accumulation of vsiRNAs in *Stat1/2^−/−^* mice compared to that in *Rag1^−/−^* mice, indicating that the signaling of IFN-I, IFN-II, or IFN-III in *Rag1^−/−^* adult mice is not inhibitory to the production of vsiRNAs. Our findings thus suggest that Dicer processing of viral dsRNA replicative intermediates into vsiRNAs is distinct from that of artificial long dsRNA, which is processed into functional siRNAs only in undifferentiated cells and IFN-defective differentiated cells (21–24, 52).

We further show that infection with NoVΔB2 or NoVmB2 induced *in vivo* production of vsiRNA-RISC active to direct Ago2-mediated, vsiRNA-guided specific RNA cleavage in an *in vitro* slicing assay. We show that the target RNA slicing by vsiRNA-RISC required the base pairing of the target RNA with the 10th nucleotide of the vsiRNA. However, loss of IFN-I, -II, and -III signaling in *Stat1/2^−/−^* mice did not enhance RNA slicing by the *in vivo*-assembled vsiRNA-RISC compared to that in *Rag1^−/−^* mice with an intact IFN system. Moreover, we observed no obvious differences in Ago2-mediated RNA slicing by the endogenous miRNA-RISC isolated from *Rag1^−/−^* or *Stat1/2^−/−^* mice after either mock or NoVΔB2 infection. It is unclear why our results from the *in vivo* assembled RISC are different from an earlier study that demonstrated inhibition of Ago2-mediated RNA slicing by miRNA-RISC in human 293T cells upon activation of IFN-I signaling (30). Together, our results indicate, for the first time, that the vsiRNAs produced by adult mice in response to viral infection are biologically active in RNAi and that the IFN response is not antagonistic to either the production of the vsiRNAs or the RNA slicing activity of the *in vivo*-assembled vsiRNA-RISC.

We show that genetic suppression of RNAi in Dicer-KO and Ago2-KO MEFs as well as in Ago2-CD MEFs significantly enhanced NoV RNA1 replication and RNA3 transcription, indicating that both Dicer-mediated vsiRNA biogenesis and Ago2 slicer activity are required for antiviral RNAi. Similarly, viral suppression of RNAi by VSR-B2, effective against RNAi induced by short hairpin RNA (53), also significantly increased the accumulation of both NoV RNA1 and RNA3 in wild-type MEFs. Unlike that in wild-type MEFs, however, the replication-enhancing activity of VSR-B2 became insignificant in all of the three lines of RNAi-defective MEFs, as found previously in S. cerevisiae that lacks the RNAi pathway (43). Thus, VSR-B2 enhances viral RNA replication only in cells when antiviral RNAi is active, demonstrating that VSR-B2 acts mainly to suppress RNAi. Consistently, expression of VSR-B2 had no major effect on the induction of ISGs in MEFs, including Dicer-KO, Ago2-KO, and Ago2-CD MEFs in which B2 was expressed at high levels.

Moreover, we demonstrate potent activation of the OAS/RNase L system in Dicer-KO MEFs following NoV RNA replication in both the presence and absence of VSR-B2. Deep sequencing detected abundant virus-derived small RNAs in the Dicer-KO MEFs, which exhibit an overwhelmingly positive-strand bias without size preference and thus may correspond to the derivatives of RNase L products from single-strand RNA (ssRNA) substrates. Similar populations of viral small RNAs were also detected in MEFs and adult mice following robust NoV RNA replication in the presence of a functional VSR-B2. These findings together suggest that in contrast to the known suppression of Dicer processing of dsRNA (26, 53), the dsRNA-binding VSR-B2 does not suppress dsRNA-dependent OAS activation or subsequent RNase L-mediated degradation of ssRNAs. Perhaps, the VSR-B2-bound long dsRNA remains as an efficient activator of OAS but is poorly recognized by Dicer. Interestingly, the OAS/RNase L system was not potently activated in Ago2-KO and Ago2-CD MEFs, although both lines of RNAi-defective MEFs supported similarly robust replication of NoV RNA1 or R1ΔB2 as found in Dicer-KO MEFs, suggesting that activation of the OAS/RNase L system may be attenuated by either Dicer processing or Dicer sequestration of viral dsRNA.

Mammalian antiviral RNAi has been documented during infection of either undifferentiated cells with wild-type viruses or differentiated cells and mice with mutant viruses rendered defective in RNAi suppression (10–15). However, previous studies have shown that a range of wild-type RNA viruses do not trigger production of a dominant peak of vsiRNAs in several commonly used lines of mature mammalian cells or replicate to higher levels in human 293T cells upon Dicer inactivation (15, 54–58). These findings led to the hypothesis that antiviral RNAi may not inhibit infection of mature cells by wild-type viruses. In this work, we show that replication of wild-type NoV RNA1 in the presence of a functional VSR-B2 triggered the production of low-abundant vsiRNAs and was significantly enhanced by genetic suppression of RNAi in MEFs. These results indicate that antiviral RNAi remains partially active in MEFs despite expression of a functional VSR. Interestingly, Ago4 is also required for antiviral defense in MEFs, possibly by promoting the production of vsiRNAs or stability of vsiRNA-RISC (59). As indicated by an earlier study (12), therefore, MEFs appear to serve as a better model for antiviral RNAi than other cell culture models. Notably, low-abundant vsiRNAs were also detectable by deep sequencing in wild-type NoV-infected adult mice both before and after pan-Argonaute co-immunoprecipitation and were able to guide RNA cleavages in the vsiRNA-RISC purified *in vivo*. Our findings provide evidence for an antiviral role of the mammalian siRNA response against infection with a wild-type virus encoding a functional VSR.

Future work is necessary to develop a conditional knockout system for investigating the *in vivo* antiviral function of Dicer or Ago2 because of their essential function in animal development (5, 6). Nevertheless, several lines of evidence from this work suggest a natural antiviral function of the RNAi in adult mice. We show that expression of VSR-B2 in adult mice suppressed the production of both vsiRNAs and RNA slicing-competent vsiRNA-RISC but had no obvious effect on the function of endogenous miRNAs or the induction of the IFN-β gene and two ISGs. Notably, VSR-B2 dramatically enhanced viral load and promoted lethal NoV infection not only in the IFN-competent *Rag1^−/−^* adult mice but also in *Stat1/2^−/−^* adult mice defective in the signaling by IFN-I, -II, and -III. When VSR-B2 was rendered nonexpressing or nonfunctional, the resulting NoV mutants induced no weight loss or any other signs of disease and were largely cleared by 10 days postinfection in both *Rag1^−/−^* and *Stat1/2^−/−^* adult mice. These results suggest a key function for the RNAi response to confer protective immunity against viral infection in adult mice either intact or defective in the IFN response.

## MATERIALS AND METHODS

### Cell lines and mice.

Wild-type and Dicer-KO, Ago2-KO, and Ago2-CD mouse embryonic fibroblasts (MEFs) were described previously (42) and confirmed by genotyping PCR and sequencing in the Ding lab. C57BL/6, *Rag1*^−/−^, and *Stat2*^−/−^ mice were purchased from the Jackson Laboratory (Sacramento, CA). *Stat1*^−/−^ mice were a kind gift from Adolfo Garcia-Sastre (Icahn School of Medicine at Mount Sinai, NY). *Stat1/2*^−/−^ double-knockout mice were obtained by crossing *Stat1*^−/−^ and *Stat2*^−/−^ single-knockout mice, with the genotype verified by PCR. Animals were housed and bred in the Animal Resources Facility under specific-pathogen-free conditions according to the guidelines described under the federal Animal Welfare Regulations Act. All animal procedures were approved by the Institutional Animal Care and Use Committee at the University of California, Riverside.

### Mouse infection.

Nodamura virus (NoV) and its two mutants, NoVmB2 and NoVΔB2, were described previously (15). NoVΔB2 contains three point mutations in RNA1 to terminate B2 translation, whereas NoVmB2 expresses a mutant B2 protein defective in dsRNA binding and RNAi suppression; however, the genetic change in neither mutant virus alters the amino acid sequence of the viral replicase encoded by RNA1 or the B1 protein, which is identical in sequence to the C-terminal region of the viral replicase and is translated from RNA3. For all adult mouse infections, sex-matched 6- to 8-week-old mice were infected by intraperitoneal injection of 150 μl of NoV, NoVmB2, or NoVΔB2 virus particle suspension titrated to contain 4.5 × 10^9^ copies of the viral genomic RNA1 in 1× Dulbecco’s modified Eagle’s medium (DMEM; Gibco) supplemented with 0.3% bovine serum albumin (BSA; Invitrogen). Littermates of the same sex were randomly assigned to experimental groups. For survival and body weight change experiments, mock- or virus-infected mice were observed for 4 to 6 weeks postinfection. Virus inoculations were performed under anesthesia, and all efforts were made to minimize animal suffering. Virion preparations from NoV-, NoVmB2-, or NoVΔB2-infected adult mice were used to inoculate suckling C57BL/6 mice, and systemic virus infection in the suckling mice was verified by quantitative RT-PCR as described previously (15, 37, 60). Nucleotide sequencing of the progeny NoVmB2 and NoVΔB2 obtained from *Rag1*^−/−^ adult mice at 5 days postinjection indicated no reversal of the introduced mutations after *in vivo* infection.

### *In vitro* transcription and electroporation.

Full-length infectious cDNA clones of NoV and NoVΔB2 were described previously (15). Transcripts of NoV RNA1 and RNA1 ΔB2 (R1ΔB2) were transcribed *in vitro* by T7 RNA polymerase with the kit mMESSAGE mMACHINE (AM1344; Invitrogen) according to the manufacturer’s instructions. After DNase I digestion, RNAs were purified by TRIzol reagent (Sigma) and analyzed by denaturing agarose gel electrophoresis and NanoDrop measurement. Wild-type and mutant MEF cell lines as described previously (42) were cultured in DMEM supplemented with 10% fetal bovine serum, 2 mM l-glutamine (Gibco), and 1× Anti-Anti (Gibco). Electroporation of MEFs with RNA1 transcripts was conducted essentially as described previously (61). Briefly, 5 million cells were collected and resuspended in 300 μl of ice-cold 1× phosphate-buffered saline PBS for each electroporation. Immediately after mixing with 3 μg of RNA1 transcripts in a 2-mm-gap electroporation cuvette (Bio-Rad), cells were subjected to electroporation with a Gene Pulser II electroporation system (Bio-Rad) under the conditions of 300 V, 75 μF, two pulses. After electroporation, cells were recovered at room temperature for 10 min before being resuspended in complete cell culture medium and split into 6-cm cell culture dishes. Cells were lysed with TRIzol reagent or 1× radioimmunoprecipitation assay (RIPA) buffer at designed time points and stored at –80°C for cellular total RNA extraction or protein quantification and Western blot.

### RNA extraction.

Immediately after mouse euthanization, the hind limb skeletal muscle tissues were collected in Eppendorf tubes with metal beads, flash-frozen in liquid nitrogen, and then stored at −80°C. For RNA extraction, 1 ml of cold TRIzol reagent was added to each tube and homogenized using TissueLyzer II (Qiagen). After removal of cell debris, total RNA was extracted by TRIzol reagent. Total RNA was also extracted from MEFs by TRIzol reagent.

### Detection of the viral low- and high-molecular-weight RNAs.

Northern blotting detection of the viral low- and high-molecular-weight RNAs was conducted as described (15). Briefly, 20 μg of total RNA extracted from the limb muscle tissues or MEFs cells were analyzed for the accumulation of the virus-derived siRNAs and mouse microRNA-22-3p. The probe used for vsiRNA detection in MEFs cells was the same as described previously (15). The probe used for vsiRNA detection in adult mouse muscle tissue was a mixture of two synthetic ^32^P-labeled locked nucleic acid (LNA) oligonucleotides purchased from Exiqon (Woburn, MA) according to small RNA deep sequencing. These LNA probes corresponded to nucleotides 1 to 25 of NoV RNA1 and nucleotides 3155 to 3179 of the negative-strand NoV RNA1 (see Table S2 in the supplemental material). For the detection of the viral genomic RNA1 and subgenomic RNA3, approximately 4 μg of total RNA was analyzed using a ^32^P α-dCTP-labeled (PerkinElmer) DNA fragment corresponding to the B2 coding regions of RNA1 and RNA3.

10.1128/mBio.03278-19.2 TABLE S2 List of RT-qPCR primers. Download Table S2, DOCX file, 0.1 MB. Copyright © 2020 Han et al. 2020 Han et al. This content is distributed under the terms of the Creative Commons Attribution 4.0 International license.

### Western blot analysis.

Western blot detection of NoV and mouse proteins was carried out as described previously with minor modifications (15). Protein lysates of adult mouse hind limb skeletal muscle tissues were obtained by homogenization in 1× RIPA buffer (Cell Signaling) supplemented with cOmplete Protease Inhibitor Cocktail (Roche) and phosphatase inhibitor cocktail PhosStop (Roche) using TissueLyzer II (Qiagen). Protein lysates of MEFs cells were prepared by directly dissolving cells into 1× RIPA buffer. NoV B2 and coat protein (CP) proteins were probed with house-made polyclonal rabbit antibodies. Detection of glyceraldehyde 3-phosphate dehydrogenase (GAPDH) by a mouse monoclonal anti-GAPDH antibody (MA5-15738; Invitrogen) served as the loading control.

### Quantitative RT-PCR.

One microgram of total RNA was used for cDNA synthesis with an iScript cDNA Synthesis kit (Bio-Rad). The cDNA products were subjected to quantitative PCR by using iQ SYBR green Supermix (Bio-Rad). Primers for virus RNAs or host mRNAs are list in Table S2. The detection of NoV RNA1 using β-actin mRNA as the internal reference was as described previously (15). Transcriptional induction of IFN-β, ISG15, and RIG-I was analyzed by the comparative threshold cycle (2^ΔΔ^*^CT^*) method (62) using β-actin mRNA and mock transfection or infection samples as controls.

### Immunoprecipitation.

Two milligrams of muscle tissue lysates in 1 ml RIPA buffer was precleared by incubation with 30 μl of protein A/G PLUS-agarose beads (Santa Cruz Biotechnology) for 30 min. Precleared lysates were then incubated with 20 μl of anti-pan Ago antibody (MABE56; Millipore) together with 40 μl protein A/G PLUS-agarose beads for 3 h at 4°C. After washing 3 times, the immunoprecipitates were used for the *in vitro* cleavage assay or small RNA library construction.

### *In vitro* cleavage assay.

The assay was performed as described previously (63) with minor modifications. The immunoprecipitates obtained with either anti-pan Ago antibody or normal mouse IgG (12-371; Sigma) as described above were washed three times with 1× wash buffer and two additional times in 1× PBS. The resulting beads were mixed with 2 μl of 1 nM ^32^P-labeled RNA substrate in 1× cleavage buffer (63) and inoculated at 30°C for 2 h before RNA extraction with TRIzol reagent. Final RNA extracts were analyzed by 15% denaturing polyacrylamide gel electrophoresis and exposed to a phosphorimager. RNA ladder (10 to 150 bp, AM7778; Invitrogen) was used as a size marker.

### Construction and analysis of small RNA libraries.

Libraries of small RNAs were constructed as described previously (15) from total RNA extracted from MEFs and hind limb muscular tissues either without or with co-immunoprecipitation by anti-pan Ago antibody (Millipore). RNA reads in 18 to 28 nucleotides were mapped to the virus and mouse genomes and analyzed as described previously (15): Mus musculus mature miRNAs and miRNA precursors, database miRBase 19; Mus musculus whole genome, the September 2017 (GRCm38.p6) assembly of the mouse genome (mm10; Genome Reference Consortium Mouse Build 38 [GCA_000001635.8]).

### Quantification and statistical analysis.

Unpaired Student's *t* test was used for statistical analysis of RT-qPCR data. Mouse body weight changes were analyzed by two-way analysis of variance (ANOVA) followed by a *post hoc* multiple-comparison test. Comparison of survival curves was conducted by using a log rank (Mantel-Cox) test. All statistical analyses and graph making were performed by using GraphPad Prism version 7.04.

### Data availability.

The accession number for the small RNA libraries listed in Table S1 is NCBI BioProject PRJNA529951.

10.1128/mBio.03278-19.3 TABLE S3 List of conventional PCR primers. Download Table S3, DOCX file, 0.1 MB. Copyright © 2020 Han et al. 2020 Han et al. This content is distributed under the terms of the Creative Commons Attribution 4.0 International license.

10.1128/mBio.03278-19.4 TABLE S4 List of Northern blot probes. Download Table S4, DOCX file, 0.1 MB. Copyright © 2020 Han et al. 2020 Han et al. This content is distributed under the terms of the Creative Commons Attribution 4.0 International license.
