# Supplementary material for: Mechanism and Function of Antiviral RNA Interference in Mice
Source: mBio. 2020 Aug 4;11(4):e03278-19. doi: 10.1128/mBio.03278-19 (PMC7407090; doi:10.1128/mBio.03278-19)
Supplement: TABLE S1 [file mBio.03278-19-st001.docx]

**Table S1 Contents and properties of the small RNA libraries.**

| Library | Mapped reads^1^  (of total reads) | Virus reads  (18-28 nt) | Pre-miR hairpins reads^2^ (% of the mapped reads) | | | vsiRNAs (21- to 23-nt) | | | | | | |
| --- | --- | --- | --- | --- | --- | --- | --- | --- | --- | --- | --- | --- |
|  |  |  |  |  |  | % of  mapped reads | % of total virus reads | | (+)-strand  (%) | | 1U  (%) | |
| NoVRNA1: Total, MEFs-wt, 24 hpt (2018122528, Fig.1) | 7,570,218 (91.2%) | 55,268 | 68.1 | | | 0.30 | 41.3 | | 86.1 | | 22.1 | |
| NoVRNA1∆B2: Total, MEFs-wt, 24 hpt (2018122529, Fig.1) | 9,877,277 (91.1%) | 29,778 | 66.7 | | | 0.20 | 66.4 | | 48.3 | | 28.7 | |
| NoVRNA1: Total, Dicer-KO MEFs, 24 hpt (2018122524, Fig.1) | 12,258,722 (80.8%) | 6,731,879 | 4.0 | | | 9.95 | 18.1 | | 98.2 | | 14.9 | |
| NoVRNA1∆B2: Total, Dicer-KO MEFs, 24 hpt (2018122526, Fig.1) | 13,706,142 (83.2%) | 2,666,556 | 5.2 | | | 6.42 | 33.0 | | 96.7 | | 14.0 | |
| NoVRNA1: Total, Ago2-KO MEFs, 24 hpt (2018122515, Fig.1) | 12,699,007 (86.0%) | 1,081,983 | 55.2 | | | 3.12 | 36.6 | | 90.9 | | 17.0 | |
| NoVRNA1∆B2: Total Ago2-KO MEFs, 24 hpt (2018122516, Fig.1) | 9,082,066 (80.6%) | 618,156 | 57.2 | | | 5.26 | 77.2 | | 44.0 | | 28.8 | |
| NoVRNA1: Total, Ago2-CD MEFs, 24 hpt (2018122512, Fig.1) | 9,675,633 (89.3%) | 1,477,102 | 48.3 | | | 6.05 | 39.6 | | 98.1 | | 20.0 | |
| NoVRNA1∆B2: Total, Ago2-CD MEFs, 24 hpt (2018122513, Fig.1) | 9,599,651 (87.1%) | 531,660 | 50.2 | | | 3.76 | 67.8 | | 61.5 | | 31.3 | |
| NoVRNA1: Total, MEFs-wt, 25 hpt (2017093001, Fig.S1) | 28,220,242 (90.4%) | 45,352 | 68.3 | | | 0.07 | 42.3 | | 84.9 | | 22.7 | |
| NoVRNA1∆B2: Total, MEFs-wt, 25 hpt (2017093002, Fig.S1) | 12,297,571 (91.2%) | 4,976 | 69.0 | | | 0.02 | 61.3 | | 50.9 | | 28.3 | |
| NoVRNA1: Total, Dicer-KO MEFs, 24 hpt (2018122523, Fig.S1) | 12,513,682 (80.5%) | 6974939 | 4.2 | | | 8.86 | 15.9 | | 98.2 | | 15.1 | |
| NoVRNA1∆B2: Total, Dicer-KO MEFs, 25 hpt, (2018122525, Fig.S1) | 16,678,035 (80.8%) | 3473187 | 5.4 | | | 6.55 | 31.3 | | 96.7 | | 13.7 | |
| NoVRNA1: Total, Ago2-KO MEFs, 25 hpt (2017093003, Fig.S1) | 18,142,092 (86.9%) | 979,289 | 54.2 | | | 1.65 | 30.5 | | 92.5 | | 15.8 | |
| NoVRNA1∆B2: Total Ago2-KO MEFs, 25 hpt (2017093004, Fig.S1) | 39,442,432 (82.5%) | 1,571,621 | 56.3 | | | 2.75 | 68.9 | | 52.2 | | 30.2 | |
| NoVRNA1: Total, Ago2-CD MEFs, 25 hpt (2017093005, Fig.S1) | 38,043,817 (91.0%) | 1,818,663 | 63.2 | | | 1.64 | 34.3 | | 98.1 | | 17.6 | |
| NoVRNA1∆B2: Total, Ago2-CD MEFs, 25 hpt (2017093006, Fig.S1) | 34,905,477 (88.0%) | 868,083 | 62.7 | | | 1.50 | 60.3 | | 62.2 | | 30.9 | |
| NoV∆B2: Total, *Rag1*^-/-^ adult, 5 dpi, #1(2018071602, Fig.3) | 10,652,104 (88.4%) | 324,401 | 63.9 | | | 2.64 | 86.6 | | 71.1 | | 65.1 | |
| NoVmB2: Total, *Rag1*^-/-^ adult, 5 dpi (2017093001, Fig.3) | 29,316,649 (92.2%) | 738,909 | 66.9 | | | 2.01 | 79.6 | | 69.1 | | 56.5 | |
| Library | Mapped reads^1^  (of total reads) | Virus reads  (18-28 nt) | | Pre-miR hairpins reads ^2^ (% of the mapped reads) | vsiRNAs (21- to 23-nt) | | | | | | |  |
|  |  |  |  |  | % of the mapped reads | | % of total virus reads | (+)-strand (%) | | 1U  (%) | |  |
| NoVmB2: Ago-IP, *Rag1*^-/-^ adult, 5 dpi (2017093002, Fig.3) | 45,758,598 (94.6%) | 754,867 | | 73.3 | 1.46 | | 88.5 | 79.7 | | 74.5 | |  |
| NoV: Total, *Rag1*^-/-^ adult, 5 dpi (2017093003, Fig.3) | 64,139,915 (94.0%) | 2,039,794 | | 64.4 | 1.25 | | 39.2 | 78.7 | | 16.0 | |  |
| NoV: Ago-IP, *Rag1*^-/-^ adult, 5 dpi (2017093004, Fig.3) | 32,982,074 (95.4%) | 96,334 | | 72.1 | 0.10 | | 35.4 | 90.4 | | 23.7 | |  |
| NoV: Total, C57BL/6 adult, 5dpi (2018122503, Fig.S5) | 12,741,338 (93.0%) | 11,224 | | 71.3 | 0.02 | | 25.6 | 86.0 | | 20.0 | |  |
| NoV∆B2: Total, C57BL/6 adult, 5dpi, #1 (2018122507, Fig.S5) | 12,044,757 (92.8%) | 7,529 | | 72.0 | 0.04 | | 68.2 | 59.5 | | 39.7 | |  |
| NoV: Total, Stat1/2^-/-^ adult, 5dpi, (2018102004, Fig.S5) | 10,393,498 (89.1%) | 694,793 | | 47.21 | 2.30 | | 30.6 | 89.7 | | 15.9 | |  |
| NoV∆B2: Total, Stat1/2^-/-^ adult, 5dpi, (2018102008, Fig.S5) | 10,113,934 (92.5%) | 3,881 | | 66.8 | 0.04 | | 87.8 | 85.1 | | 77.9 | |  |

^1^ Mapped reads include all validated reads in the sequenced library mapped to both the mouse and virus genome.

^2^ Pre-miR hairpins include mature microRNAs, microRNA* and imprecise products.

**Table S2. List of RT-qPCR primers**

| **RT-qPCR primers** | **Sequence** |
| --- | --- |
| ISG15-forward | 5’ TGGTACAGAACTGCAGCGAG 3’ |
| ISG15-reverse | 5’ CAGCCAGAACTGGTCTTCGT 3’ |
| IFN-β-forward | 5’ AAGAGTTACACTGCCTTTGCCATC 3’ |
| IFN-β-reverse  RIG-I-forward  RIG-I-reverse | 5’ CACTGTCTGCTGGTGGAGTTCATC 3’  5’ GAGAGTCACGGGACCCAC T 3’  5’ CGG TCTTAGCATCTCCAA CG 3’ |
| NoV-forward | 5’ CCGTTCATGGCTTACACCTT 3’ |
| NoV-reverse | 5’ GCACCAGTCCCAAACTTCAT 3’ |
| β-actin- forward | 5’ ATT GGC AAC GAG CGG TTC C 3’ |
| β-actin- reverse | 5’ AGC ACT GTG TTG GCA TAG AGG 3’ |

**Table S3. List of Conventional PCR Primers**

| **Conventional PCR primers ^1^** | **Sequence** |
| --- | --- |
| RAG1 wildtype -forward | 5’ TCT GGA CTT GCC TCC TCT GT 3’ |
| RAG1 common-reverse | 5’ CAT TCC ATC GCA AGA CTC CT 3’ |
| RAG1 mutant -forward | 5’ TGG ATG TGG AAT GTG TGC GAG 3’ |
| STAT1 wildtype -reverse | 5’ GCTGGTGGACCTGCTCCAGGAACTT 3’ |
| STAT1 common-forward | 5’AGGGAATGTGTGATGGGTCAGGGTGATAAATAC 3’ |
| STAT1 mutant -reverse | 5’ GGGAGGATTGGGAAGACAATAGCAGGCATG 3’ |
| STAT2 wildtype -forward | 5’ CCTTCTATCGCCTTCTTGACGAGTTCTTCTGA 3’ |
| STAT2 common-reverse | 5’ AGC AGG GCT CAA ACT CAC AG 3’ |
| STAT2 mutant -forward | 5’ GACCAGGATCTCCTCCACCCTCTGCGG 3’ |

^1^ Expected length:

RAG1: wildtype 192 bp, mutant 197 bp

STAT1: wildtype 560 bp, mutant 262 bp

STAT2: wildtype 158 bp, mutant 520 bp

**Table S4. List of Northern blot probes**

| **Northern blot probes** | **Sequence** |
| --- | --- |
| Probes for VsiRNA detection in adult mice (Mixture of two LNA oligoes) | 5’ GTATTGAATCCAAAACTCAAAATGC 3’  5’ CCGTTGATGATTGTCTCGTAGTTCA 3’ |
| miR-22-3P probe | 5’ ACAGTTCTTCAACTGGCAGCTT 3’ |
| U6 probe | 5’GAATTTGCGTGTCATCCTTGCGCAGGGGCCATGCTAA 3’ |
| 18S rRNA 5’ probe | TAATCTTTGAGACAAGCATATGCTACCTGGCAGGATCAACCAGGT |
| 18S rRNA 3’ probe | TTAATGATCCTTCCGCAGGTTCACCTACGGAAACCTTGTTACGAC |
| 28S rRNA 5’ probe | AATATGCTTAAATTCAGCGGGTCGCCACGTCTGATCTGAGGTCGCG |
| 28S rRNA 3’ probe | GAAAGCCCGCAGAGACAAACCCTTGTGTCGAGGGCTGACTTTCAA |

Underlined nucleotides are LNA nucleotides.
