# Supplementary figures and images for: Mechanism and Function of Antiviral RNA Interference in Mice
Source: mBio. 2020 Aug 4;11(4):e03278-19. doi: 10.1128/mBio.03278-19 (PMC7407090; doi:10.1128/mBio.03278-19)

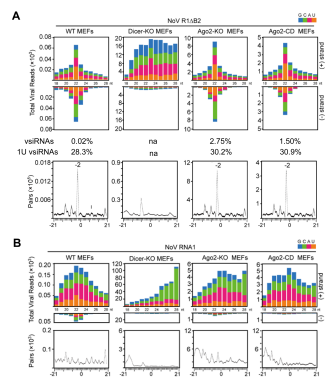

Supplement: FIG S1 [file mBio.03278-19-sf001.pdf]

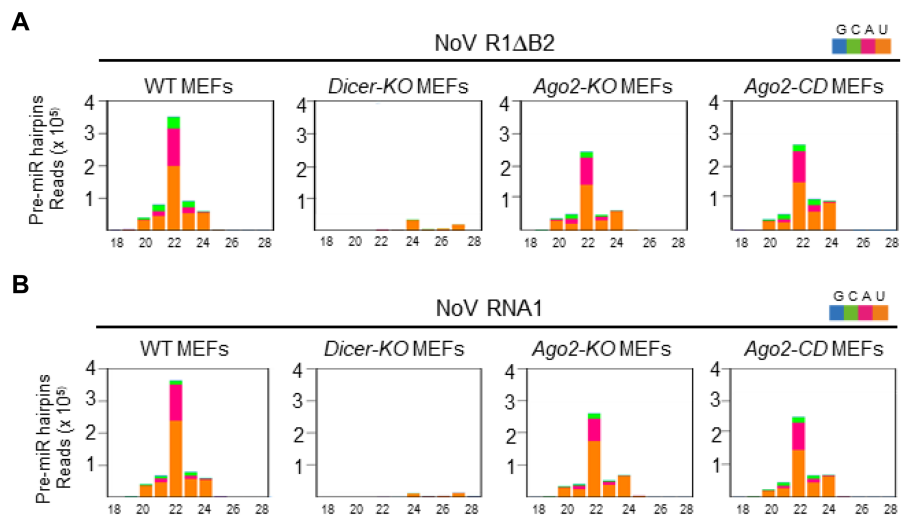

Supplement: FIG S2 [file mBio.03278-19-sf002.pdf]

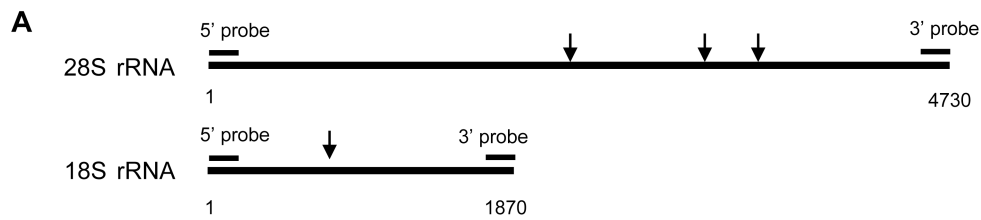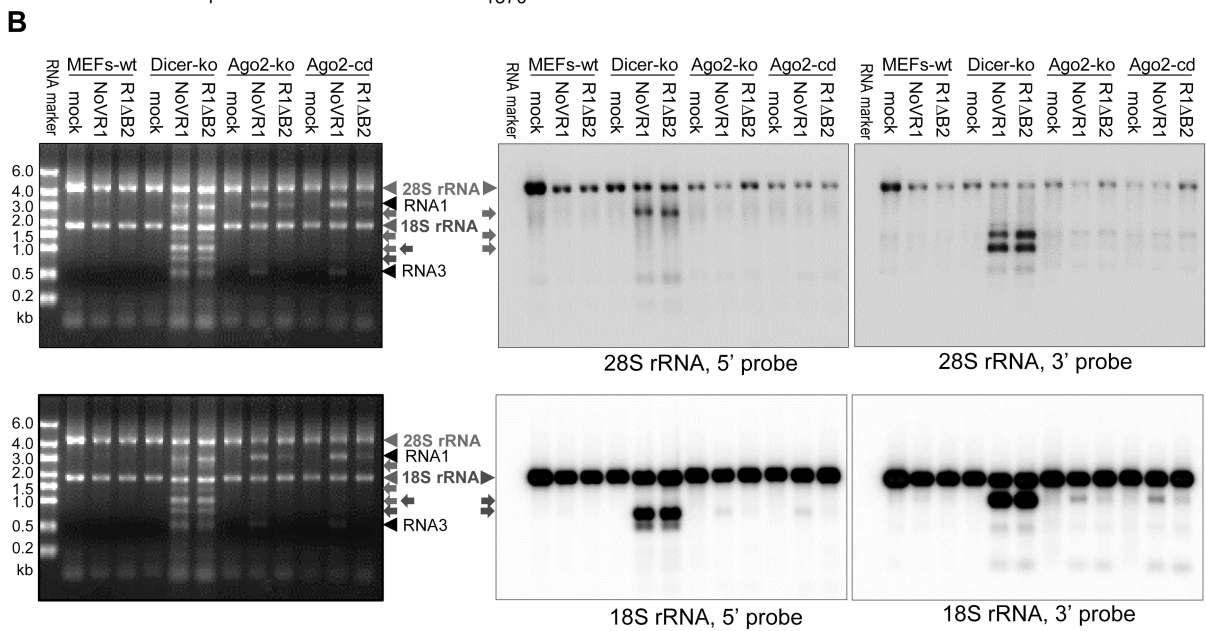

Supplement: FIG S3 [file mBio.03278-19-sf003.pdf]

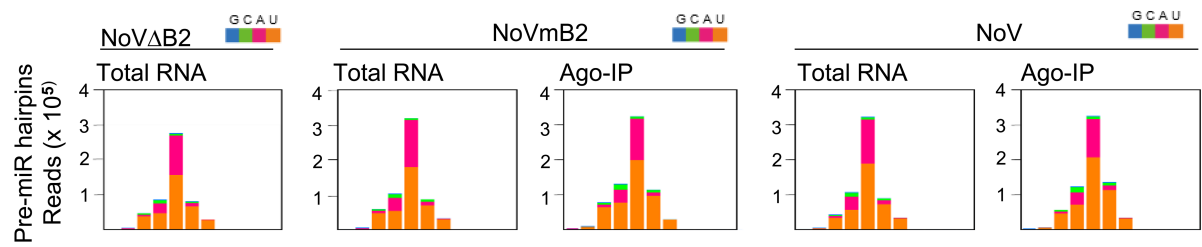

Supplement: FIG S4 [file mBio.03278-19-sf004.pdf]

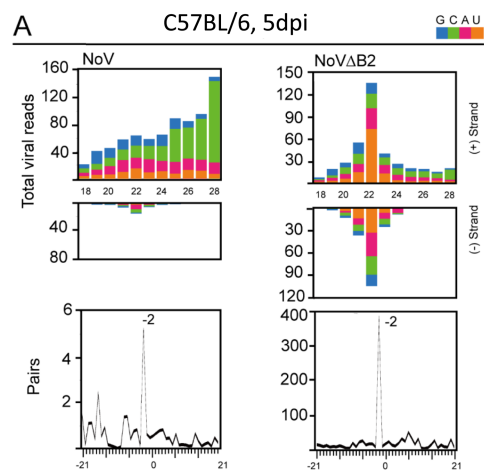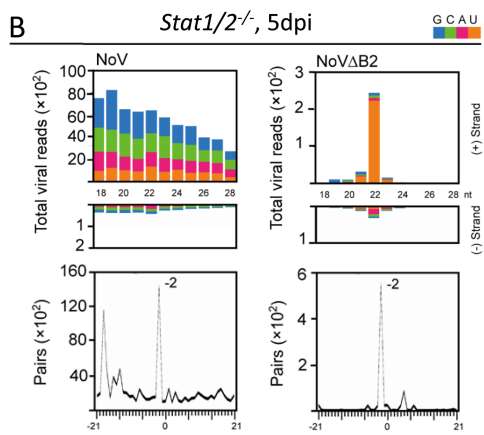

Supplement: FIG S5 [file mBio.03278-19-sf005.pdf]
