# Supplementary material for: Mechanism and Function of Antiviral RNA Interference in Mice
Source: mBio. 2020 Aug 4;11(4):e03278-19. doi: 10.1128/mBio.03278-19 (PMC7407090; doi:10.1128/mBio.03278-19)
Supplement: TABLE S2 [file mBio.03278-19-st002.docx]

**Table S2. List of RT-qPCR primers**

| **RT-qPCR primers** | **Sequence** |
| --- | --- |
| ISG15-forward | 5’ TGGTACAGAACTGCAGCGAG 3’ |
| ISG15-reverse | 5’ CAGCCAGAACTGGTCTTCGT 3’ |
| IFN-β-forward | 5’ AAGAGTTACACTGCCTTTGCCATC 3’ |
| IFN-β-reverse  RIG-I-forward  RIG-I-reverse | 5’ CACTGTCTGCTGGTGGAGTTCATC 3’  5’ GAGAGTCACGGGACCCAC T 3’  5’ CGG TCTTAGCATCTCCAA CG 3’ |
| NoV-forward | 5’ CCGTTCATGGCTTACACCTT 3’ |
| NoV-reverse | 5’ GCACCAGTCCCAAACTTCAT 3’ |
| β-actin- forward | 5’ ATT GGC AAC GAG CGG TTC C 3’ |
| β-actin- reverse | 5’ AGC ACT GTG TTG GCA TAG AGG 3’ |
