# Supplementary material for: Mechanism and Function of Antiviral RNA Interference in Mice
Source: mBio. 2020 Aug 4;11(4):e03278-19. doi: 10.1128/mBio.03278-19 (PMC7407090; doi:10.1128/mBio.03278-19)
Supplement: TABLE S3 [file mBio.03278-19-st003.docx]

**Table S3. List of Conventional PCR Primers**

| **Conventional PCR primers^1^** | **Sequence** |
| --- | --- |
| RAG1 wildtype -forward | 5’ TCT GGA CTT GCC TCC TCT GT 3’ |
| RAG1 common-reverse | 5’ CAT TCC ATC GCA AGA CTC CT 3’ |
| RAG1 mutant -forward | 5’ TGG ATG TGG AAT GTG TGC GAG 3’ |
| STAT1 wildtype -reverse | 5’ GCTGGTGGACCTGCTCCAGGAACTT 3’ |
| STAT1 common-forward | 5’AGGGAATGTGTGATGGGTCAGGGTGATAAATAC 3’ |
| STAT1 mutant -reverse | 5’ GGGAGGATTGGGAAGACAATAGCAGGCATG 3’ |
| STAT2 wildtype -forward | 5’ CCTTCTATCGCCTTCTTGACGAGTTCTTCTGA 3’ |
| STAT2 common-reverse | 5’ AGC AGG GCT CAA ACT CAC AG 3’ |
| STAT2 mutant -forward | 5’ GACCAGGATCTCCTCCACCCTCTGCGG 3’ |

^1^Expected length of the PCR product:

RAG1: wildtype 192 bp; mutant 197 bp

STAT1: wildtype 560 bp; mutant 262 bp

STAT2: wildtype 158 bp; mutant 520 bps
