# Supplementary material for: Mechanism and Function of Antiviral RNA Interference in Mice
Source: mBio. 2020 Aug 4;11(4):e03278-19. doi: 10.1128/mBio.03278-19 (PMC7407090; doi:10.1128/mBio.03278-19)
Supplement: TABLE S4 [file mBio.03278-19-st004.docx]

**Table S4. List of Northern blot probes**

| **Northern blot probes** | **Sequence** |
| --- | --- |
| ^1^Probes for vsiRNA detection in adult mice by a mixture of two LNA oligoes | 5’ GUAUUGAAUCCAAAACUCAAAAUGC 3’  5’ CCGUUGAUGAUUGUCUCGUAGUUCA 3’ |
| miR-22-3P probe | 5’ ACAGTTCTTCAACTGGCAGCTT 3’ |
| U6 probe | 5’ GAATTTGCGTGTCATCCTTGCGCAGGGGCCATGCTAA 3’ |
| 18S rRNA 5’ probe | 5’ TAATCTTTGAGACAAGCATATGCTACCTGGCAGGATCAACCAGGT 3’ |
| 18S rRNA 3’ probe | 5’ TTAATGATCCTTCCGCAGGTTCACCTACGGAAACCTTGTTACGAC 3’ |
| 28S rRNA 5’ probe | 5’ AATATGCTTAAATTCAGCGGGTCGCCACGTCTGATCTGAGGTCGCG 3’ |
| 28S rRNA 3’ probe | 5’ GAAAGCCCGCAGAGACAAACCCTTGTGTCGAGGGCTGACTTTCAA 3’ |

^1^Underlined nucleotides are LNA nucleotides.
